# Sex Differences in Immune Cell Infiltration and Hematuria in SCI-Induced Hemorrhagic Cystitis

**DOI:** 10.3390/pathophysiology30030023

**Published:** 2023-07-11

**Authors:** Hadi Askarifirouzjaei, Leila Khajoueinejad, Elena Wei, Sruti Cheruvu, Carlos Ayala, Ning Chiang, Thomas Theis, Dongming Sun, Mehdi Fazeli, Wise Young

**Affiliations:** 1 Keck Center for Collaborative Neuroscience, Department of Cell Biology and Neuroscience, Rutgers University, Piscataway, NJ 08554, USA; 2 Memorial Sloan Kettering Cancer Center, New York, NY 10065, USA; 3 Department of Pharmacology, School of Veterinary Medicine, Shiraz University, Shiraz 71345, Iran; 4 Weill Cornell Medical College, New York, NY 10065, USA

**Keywords:** bladder, spinal cord injury, hemorrhagic cystitis, gender, neutrophil

## Abstract

Rats manifest a condition called hemorrhagic cystitis after spinal cord injury (SCI). The mechanism of this condition is unknown, but it is more severe in male rats than in female rats. We assessed the role of sex regarding hemorrhagic cystitis and pathological chronic changes in the bladder. We analyzed the urine of male and female Sprague-Dawley and Fischer 344 rats after experimental spinal cord contusion, including unstained microscopic inspections of the urine, differential white blood cell counts colored by the Wright stain, and total leukocyte counts using fluorescent nuclear stains. We examined bladder histological changes in acute and chronic phases of SCI, using principal component analysis (PCA) and clustered heatmaps of Pearson correlation coefficients to interpret how measured variables correlated with each other. Male rats showed a distinct pattern of macroscopic hematuria after spinal cord injury. They had higher numbers of red blood cells with significantly more leukocytes and neutrophils than female rats, particularly hypersegmented neutrophils. The histological examination of the bladders revealed a distinct line of apoptotic umbrella cells and disrupted bladder vessels early after SCI and progressive pathological changes in multiple bladder layers in the chronic phase. Multivariate analyses indicated immune cell infiltration in the bladder, especially hypersegmented neutrophils, that correlated with red blood cell counts in male rats. Our study highlights a hitherto unreported sex difference of hematuria and pathological changes in males and females’ bladders after SCI, suggesting an important role of immune cell infiltration, especially neutrophils, in SCI-induced hemorrhagic cystitis.

## 1. Introduction

Traumatic injuries to the spinal cord cause considerable bladder dysfunction [1], usually attributed to the loss of neurological control of micturition, catheterization, and subsequent bacterial infections. In experimental animal spinal cord injury (SCI) models, however, some investigators [2,3,4] have noted acute hemorrhagic cystitis. While SCI patients usually show chronic mucosal ulcerations and inflammation of the bladder associated with bacterial cystitis and even cancer in the weeks or months after SCI, rats show a much more rapid and gross hematuria within a day after SCI [5]. The urinary bladder undergoes substantial anatomical and structural changes after SCI [5,6,7,8].

The urinary bladder wall Is composed of the uroepithelium, lamina propria, and detrusor muscle layers. Umbrella cells form the mucosal surface of the multi-layer urothelium. The umbrella layer undergoes a dramatic apoptotic transformation within 1 day of SCI, followed by the sloughing of the umbrella cells [5,8]. Although some of these changes are transient and may be followed by spontaneous restoration, they can cause long-term damage to the bladder structure that leaves the bladder susceptible to chronic inflammation and chronic bacterial infections [7,9,10]. Accordingly, identifying acute and chronic patterns of pathological changes should provide insights into both mechanisms and therapies of bladder damage after SCI.

Sex differences in bladder pathology after SCI are not surprising considering the marked differences in male and female urinary tract anatomy [11,12]. Because male animals have more bladder complications after SCI, many investigators have preferred to use only female SCI animal models [13]. Even though unisex SCI models are likely to introduce a sex bias [14], few investigators have studied the effects of sex on the pathophysiology of hemorrhagic cystitis after SCI [15]. Additionally, despite a growing body of literature showing sex-based differences in male and female immune responses [16,17,18], the roles of immunological differences between males and females regarding the bladder after SCI require further investigation.

Infiltrating myeloid cells, especially neutrophils, play central roles in acute neuroinflammatory responses following SCI [19,20,21,22]. Certain subsets of neutrophils may promote neuroregeneration after spinal cord injury [23]. Interestingly, large numbers of neutrophils infiltrate the bladder uroepithelium, followed by a loss of bladder integrity during the acute phase of the spinal cord injury [7]. Neutrophil infiltration and activation differ considerably between men and women in acute inflammatory states [24,25] and in SCI. Yet, no studies to date have examined post-SCI bladder neuroinflammatory responses that may leave males or females more vulnerable to inflammation or hemorrhagic cystitis.

Here, in studies of a clinically relevant and well-standardized SCI contusion model [26], we uncovered remarkable sex-related differences in hemorrhagic cystitis between male and female rats after SCI. Interestingly, we found a distinct line of apoptotic urothelial umbrella cells and the profound recruitment of neutrophils including hypersegmented neutrophils into the bladder, correlating with the severity of hematuria. In contrast to spontaneous recovery from acute hemorrhagic cystitis in male rats, substantial pathological changes occur in bladder layers in the chronic phase of both male and female rats. Together, our data revealed the extraordinary sloughing of the bladder uroepithelium with a disruption in vascular integrity very early after SCI that occurs primarily in male rats that have robust hematuria. These findings underscore the susceptibility of the bladder structure in response to SCI and highlight the importance of special bladder care in the acute phase after SCI [27].

## 2. Materials and Methods

### 2.1. Retrospective Study

All of the rats received a consistent contusion of the thoracic spinal cord using the MASCIS standard weight-drop impactor [28] that dropped a 10 g weight 12.5 mm onto T9–T10 spinal cord of two rat strains: SD and F344 (*n* = SD 27 males/33 females; F344 46 males/36 females) rats. We examined urine samples collected from 142 rats from the two rat strains. Levels of gross hematuria for 3 days were scored on a 1–4-point scale according to the intensity of the blood color (1: No hematuria, 2: Mild hematuria, 3: Moderate hematuria, 4: Severe hematuria) in male and female rats.

### 2.2. Spinal Cord Contusion Injury

The contusion model of SCI was carried out as previously described [28]. After making an incision on the skin, longitudinal incisions were made on the left and right sides of the spinous processes muscles. The paravertebral muscles were then pulled away from the spine, without any further incision or damage to the muscles. Lamina was removed to expose the dorsal surface of the spinal cord. Stabilization clamps were placed at the posterior spinous processes of the vertebrae to support the vertebral column during impact. F344 rats (half male and half female) were contused with a Multicenter Animal Spinal Cord Injury Study (MASCIS) impactor that drops a 10 g weight 12.5 mm onto the T9–T10 spinal cord exposed by laminectomy. After injury, the muscles were sutured in layers, and the skin was closed with metal wound clips. Rats that received laminectomy without SCI served as controls for the effects of anesthesia and laminectomy without contusion. After surgery, we placed the rats on heating pads (37 °C) and observed them until they were stable and awake before returning them to their home cage. Pre- and post-operatively, the rats were housed in a temperature- (21 ± 1 °C) and humidity-controlled environment with a 12 h dark/light phase. The rats received ad libitum access to a certified rodent diet and water over the study period, and each rat box housed one spinal-injured rat. The Rutgers Animal Care and Facilities Committee (equivalent to the Institutional Animal Care and Use Committee) approved all the animal use and care procedures (No 99-025).

### 2.3. Urinalysis

We expressed the bladders manually and collected urine in sterile tubes. Urine samples with mild to no gross hematuria were centrifuged at 3000 RPM for 7 min, the supernatant was poured off, and the resuspended sediment was examined by a light microscope (Zeiss, Axiophot; Jena, Germany) under high-power magnification (HPF). Urine samples with moderate to severe macroscopic hematuria were examined directly without centrifugation. Microscopic red blood cell (RBC) counts of unstained urine in male and female rats were averaged for 3 days after SCI.

### 2.4. Urine Analysis: Wright Stain Differential

To make urine leukocyte differential counts, we spread a drop of urine over a glass slide and air-dried and stained the urine with the Wright-stain (LeukoStat, Fisher Scientific, Pittsburg, PA, USA). Urine samples with mild to no gross hematuria were centrifuged at 3000 RPM for 7 min, the supernatant was poured off, and the resuspended sediment was used for blood smear preparation. Leukocyte differential counts were determined using a 40× objective lens of a light microscope (Zeiss, Axiophot; Jena, Germany). All slides were blinded, and 200 leukocytes in the monolayer of the smear were manually counted by two experienced investigators. The differential count measures the percentages of each type of leukocyte present.

### 2.5. Urine Analysis: Total Leukocyte Count

For the total leukocyte count, we stained each uncentrifuged urine sample with the fluorescent dyes acridine orange and propidium iodide and counted the total WBC with a Cellometer (Nexcelom, Lawrence, MA, USA), an automated cell counter. The quantification was optimized for the volume and shape for the urine analysis chamber. All cell counts were performed in duplicate.

### 2.6. Tissue Processing for Histological Evaluation

To characterize the histological bladder changes in acute and chronic phases after SCI, we cut transverse paraffin sections of the bladder from rats euthanized 24 h and 6 weeks after SCI [29]. The rats were deeply anesthetized with injections of ketamine (80 mg/kg) and xylazine (10 mg/kg), followed by transcardial perfusion with saline containing 10 U/mL heparin and then 4% of paraformaldehyde. The bladder was then removed and left in 4% paraformaldehyde overnight at 4 °C. After fixation, the tissues were dehydrated with a graded series of ethanol solutions, cleared in xylene, and embedded in paraffin blocks for sectioning. Bladder samples were sectioned transversally with a microtome at a 5 μm thickness and mounted on slides.

### 2.7. Hematoxylin and Eosin Staining

To visualize the bladder structure during the acute phase, we cut paraffin sections and stained with hematoxylin and eosin [30]. Paraffin sections were deparaffinated. To stain the nuclei, the sections were incubated in Gill Hematoxylin (Sigma, St. Louis, MO, USA) for 10 min, rinsed in running tap water, differentiated in Bluing reagent, rinsed in distilled water followed by 95% ethanol, counterstained in Eosin Y solution (Sigma, St. Louis, MO, USA) for 20 s, rinsed in distilled water, differentiated in 95% ethanol, rinsed in 100% ethanol, cleared with xylene, and cover-slipped with mounting media.

### 2.8. Wright Staining

The bladder sections were deparaffinized and then rinsed in distilled water. The sections were stained with Wright stain (Sigma, St. Louis, MO, USA), rinsed in distilled water, and cover-slipped with mounting media.

### 2.9. Masson’s Trichrome Staining

The bladder sections were deparaffinized and then washed in distilled water. The sections were re-fixed in Bouin’s solution for 1 h at 56 °C, rinsed in running tap water, and then stained with Weigert’s iron hematoxylin solution. The sections were then rinsed in running tap water followed by a wash in distilled water, stained with Biebrich scarlet acid fuchsin, washed in distilled water, differentiated in phosphomolybdic-phosphotungstic acid solution, and stained with aniline blue solution. The sections were washed in distilled water and rinsed briefly in 1% acetic acid. The bladder sections were dehydrated followed by clearing in xylene and then cover-slipped with mounting media. Stained sections were photographed with a light microscope (Zeiss, Axiophot; Jena, Germany). We measured the bladder layers thickness using ImageJ software (Version 1.50b; National Institutes of Health) (accessed on 10 May 2023).

### 2.10. TUNEL Assay

Bladder paraffin sections were deparaffinized and then washed in distilled water. The sections were stained using ApopTag In Situ Apoptosis Detection Kits (EMD Millipore Corp; Billerica, MA, USA), according to the manufacturer’s instructions, and nuclei were counterstained by DAPI. The sections were digitally photographed with an epifluorescent microscope (Zeiss, Axiophot; Jena, Germany). The number of TUNEL and DAPI+ cells were counted for each image area using ImageJ software (accessed on 10 May 2023) (Version 1.50b; National Institutes of Health), and the percentage of TUNEL+ cells per DAPI+ cells was calculated.

### 2.11. Statistical Analysis

The experiments and analyses were double-blinded such that neither the experimenter nor the analyst knew the treatment nor source of the tissues being analyzed. We used GraphPad Prism version 9.0 (GraphPad Software Inc., San Diego, CA, USA) (accessed on 5 May 2023) for statistical analyses. A nonparametric statistical Mann–Whitney test or Friedman test followed by Dunn’s post hoc were used for rank-based gross hematuria comparisons. Student’s t-tests or ANOVA tests followed by Tukey’s post hoc test used other comparisons. The Shapiro–Wilk test was used to test for a normal data distribution.

Multidimensional reduction analysis was conducted using principal components analysis (PCA) with GraphPad Prism version 9.0 (GraphPad Software Inc., San Diego, CA, USA). Orthogonal transformation was applied to input variables including the thickness of the total bladder wall, detrusor muscle, connective tissue, and urothelium; the urine analysis parameters of 1 day and 2 days after injury were: the total leukocyte count, total neutrophils, total lymphocytes, and total hypersegmented neutrophils. Principal components (PC1, PC2) were identified, which explained the majority of the variance. The retention of PCs was determined by the “Kaiser Rule” (eigenvalue > 1). The length of the arrows indicates the magnitude of the loading for the variable in the loading plot.

Correlation analyses between histology and urine analysis parameters after SCI in male rats were evaluated by calculating the parametric Pearson’s correlation coefficient r, R2, and a two-tailed *p* value. A *p* value of <0.05 was considered to be statistically significant. To improve the visual representation of the correlation on a heatmap and facilitate finding groups of correlated features, we loaded the data onto Morpheus (https://software.broadinstitute.org/morpheus/) (accessed on 25 April 2023) and constructed a dendrogram of hierarchical clustering using 1-Pearson correlation. After computing the distance matrix, the parameters were grouped hierarchically according to their distances in the dendrogram.

## 3. Results

### 3.1. Hemorrhagic Cystitis with Acute Vascular Injury and Neutrophils Infiltration after SCI

**Figure 1:**
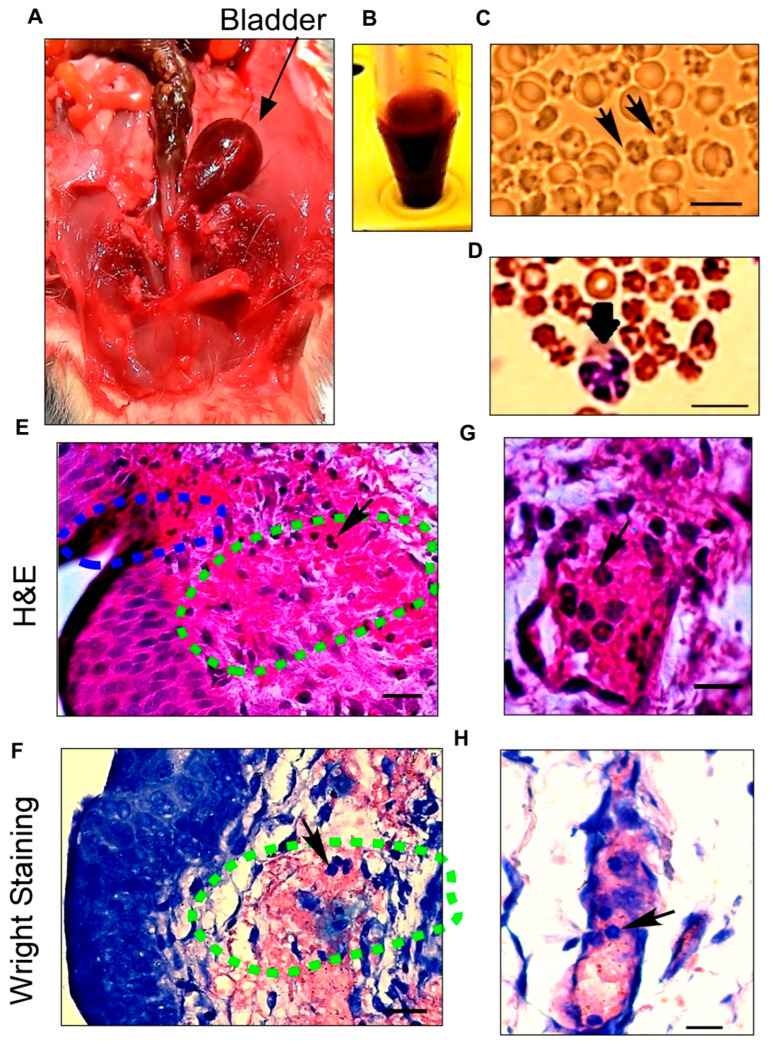
Histological characterizations of hemorrhagic cystitis show the source of severe hematuria after spinal cord injury (SCI). (**A**) Anatomical dissection of the lower abdominal area reveals a distended reddish bladder (arrow) in a male rat 1 day after SCI; (**B**) Urine samples collected 1 day after SCI illustrate gross hematuria; (**C**) Microscopic analysis of urine with an unstained method confirms the presence of crenated blood cells in the urine on the first day after SCI. Isomorphic or crenated RBCs (arrows) indicate that the cells originated from the bladder; (**D**) Wright staining on urine sample leukocytes in urine after SCI. The arrow points to a neutrophil; (**E**,**F**) Severe hemorrhagic cystitis including an ulceration (blue dashed area) in the urothelium layer and a hemorrhage with leukocyte infiltration (arrow) in the lamina propria layer (green dashed area) stained by hematoxylin and eosin; (**G**,**H**) Dilated blood vessels with increased capillary fragility are confirmed by H&E and Wright staining after SCI. Neutrophil infiltration (arrows) from blood vessels into the bladder wall during hemorrhagic cystitis can be seen using both staining methods. Scale bar = 10 μm (**C**,**D**) and 20 μm (**E**–**H**).

After dissecting the ventral lobe of the prostate to expose the urinary bladder, the gross pathological examination of the urinary bladder in the abdominal cavity in male rats revealed a cherry-colored bladder at 24 h after SCI (Figure 1A). Severe macroscopic hematuria was apparent during the first days after spinal injury (Figure 1B).

Urine analysis by the unstained method showed large numbers of isomorphic round erythrocytes combined with crenated (characterized by regular spikes) red blood cells (RBC), confirming that microscopic hematuria originated from bladder injury and not kidney damage. Morphological inspections of the RBC indicated no dysmorphic RBC and acanthocytes, confirming the non-glomerular origin of RBC in urine (Figure 1C).

The Wright staining of urine samples, in addition to showing the presence of RBC in the urine, presented dramatically increased numbers of leukocytes, especially neutrophils, in the urine samples after SCI (Figure 1D). Furthermore, microscopic sediment urine analysis, Wright staining finding, and dipstick tests indicate no sign of bacterial urinary tract infection.

The microscopic analysis of the bladder with H&E and Wright staining indicated marked hemorrhage and edema throughout the lamina propria (Figure 1E,F), with vascular dilatation and capillary changes that indicate acute vascular injury after SCI (Figure 1G,H). A severely damaged endothelium is evident, including increased vascular permeability, bleeding along with an urothelium ulceration, and endothelial erosion covered with blood. H&E staining revealed large numbers of neutrophils that had migrated to the endothelial surface and infiltrated into the lamina propria and urothelium layers. H&E and Wright staining confirmed neutrophilic hemorrhagic cystitis (Figure 1G,H).

### 3.2. Sex Differences in Hemorrhagic Cystitis in the Acute Phase after Spinal Cord Injury

**Figure 2:**
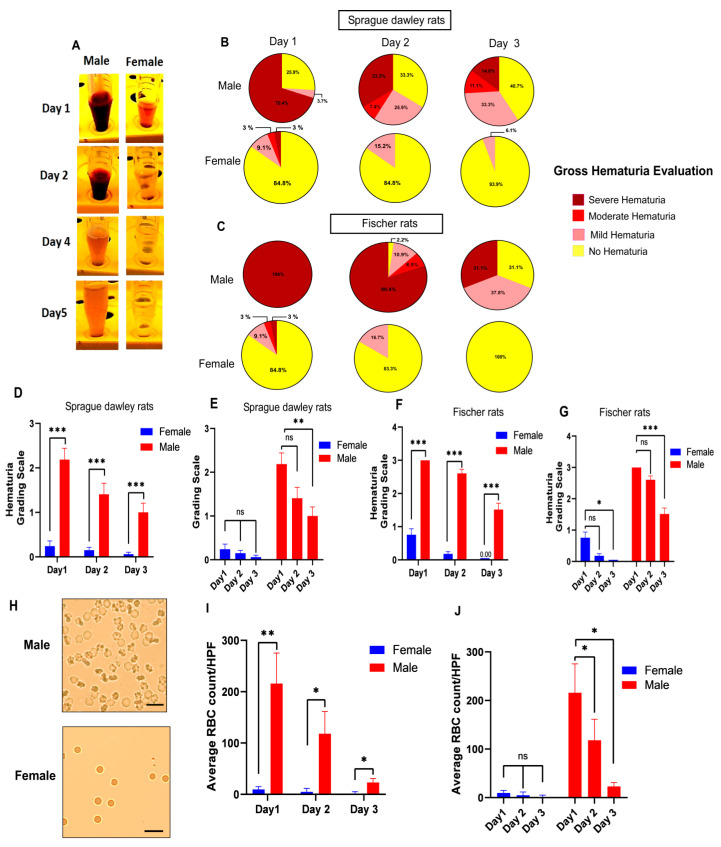
Male rats have more severe hemorrhagic cystitis compared to female rats in the acute phase after thoracic spinal cord injury in two different rat strains, Sprague-Dawley (SD) and Fischer 344 (F344). (**A**) Representative images show urine samples collected during the first five days after spinal injury. Severe hematuria in male rats compared to mild gross hematuria in female rats was observed in the first days after spinal cord injury. Representative pie charts (**B**,**C**) and bar diagrams (**D**–**G**) indicate the average percentage frequencies of different levels of gross hematuria during the 3 days following SCI. The severity of hematuria scored on a 1–4-point scale according to the intensity of blood color (1: No hematuria, 2: Mild hematuria, 3: Moderate hematuria, 4: Severe hematuria) in male and female rats. (*n* = SD 27 males/33 females; F344 46 males/36 females). Mann–Whitney test for planned multiple comparisons (**C**) or Friedman test followed by Dunn’s post hoc test for planned multiple comparisons (**D**); (**H**) Representative images showing a higher number of red blood cells (RBC) in males compared to females in urine samples collected 24 h after SCI by the unstained urine method. Scale bar = 20 µm; (**I**,**J**) Bar diagrams show the average values of the microscopic red blood cells (RBC) count of unstained urine in male and female rats (*n* = 5 males/5 females) 3 days following SCI. Student’s t-tests (day 1: df = 8, t = 3.430, ** *p* < 0.01; day 2: df = 8, t = 2.615, * *p* < 0.05; day 3: df = 8, t = 2.794, * *p* < 0.05) for planned multiple comparison (**C**) or repeated measures ANOVA test (for females comparison: F (1.016, 4.063) = 2.233, *p* = 0.2088; for males comparison: F (1.067, 4.269) = 12.83, *p* = 0.0202) followed by Tukey’s post hoc test for planned multiple comparison (**D**). * *p* ≤ 0.05, ** *p* ≤ 0.01, *** *p* ≤ 0.001, ns non-significant. Error bars are the mean ± SEM.

Severe macroscopic hematuria, especially during the first days after spinal injury in male rats, gradually cleared during the first week after injury. Mild gross hematuria in some female rats on the first day following SCI could be detected by a naked eye examination of the urine specimen (Figure 2A). The pattern and severity of macroscopic hematuria were considered in two different rat strains including SD and F344 in both males and females after SCI (Figure 2B,C). Of the 27 male SD rat urine specimens at one day after SCI, 19 (70.4%) had severe hematuria. Severe hematuria resolved 2 and 3 days following SCI in male rats, and, therefore, only nine (33.3%) and four (14.8%) had severe hematuria on day 1 and day 2, respectively. Similarly, of 46 (100%) male F344 rats with severe hematuria 24 h after SCI, macroscopic hematuria resolved in 31 (68.9%) rats in 3 days following SCI. In contrast, among 69 female rats including 33 Sprague-Dawley rats and 36 F344 rats, only 6 (16.9%) rats had severe hematuria in the urine specimen at 1 day after SCI, while macroscopic hematuria completely disappeared from 31 (93.9%) and 36 (100%) female Sprague-Dawley and F344 rats, respectively.

While both male and female rats received the same contusive spinal cord injuries, male Sprague-Dawley rats had significantly higher hematuria scores compared to female rats on day 1 after SCI (Figure 2D). Similar statistical analyses showed significantly higher hematuria scores in male Fischer 344 rats compared to female F344 rats at 24 h after SCI (Figure 2F). This pattern of more severe hematuria in both rat strains in male rats compared to female rats was also observed on day 2 and day 3 after spinal cord injury. However, the spontaneous resolution of hematuria was observed in males and females in both strains, especially on day 3 after SCI (Figure 2E,G).

To determine whether this distinct macroscopic difference in hematuria between males and females is observable at the microscopic level of examination, we used the unstained method for RBC quantification to assess the urine specimen after SCI (Figure 2H). On all three days, the comparisons of males and females based on microscopic RBC quantification showed a statistically significantly higher number of RBC in male urine samples compared to female urine samples, confirming that the pattern was observed in macroscopic urine evaluation (Figure 2I,J).

### 3.3. Apoptotic Umbrella Cells Early after SCI

**Figure 3:**
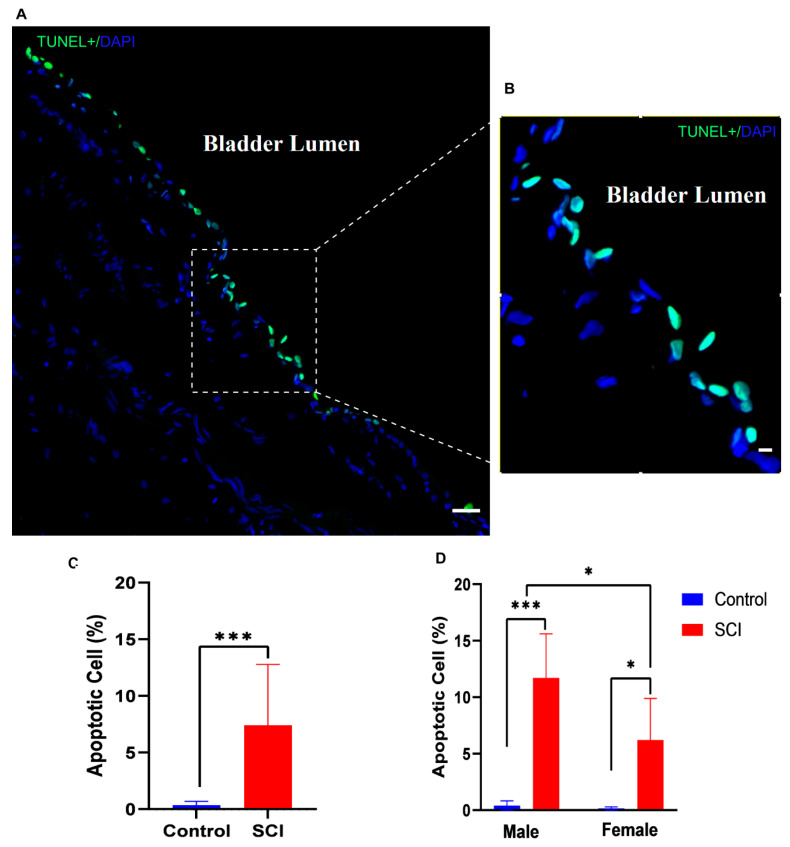
Appearance of a distinct morphological line of apoptotic cells in the bladder urothelium 24 h after spinal cord injury (SCI). (**A**) TUNEL-stained nuclei showed many cells in the bladder urothelium with apoptotic DNA fragmentation (green); (**B**) Higher-magnification image of apoptotic cells in the umbrella cell layer of the urothelium exposed to bladder lumen; (**C**) Bar diagram shows the average percentage of apoptotic positive cells in control and injured rats (df = 18, t = 4.141, *** *p* < 0.001, Student’s t test); (**D**) Bar diagram compares the percentages of apoptotic cells in male and female rats (F (1, 16) = 4.737, *p* = 0.0448 Two-way ANOVA, Tukey’s * *p* < 0.01, *** *p* < 0.001). Scale bar = 50 μm (**A**) and 20 μm (**B**). Error bars are the mean ± SD.

To define the pathological consequences of spinal cord injury in the acute phase to show it could contribute to hemorrhagic cystitis, we stained for apoptotic cells in the bladder using the TUNEL method [31]. Positive signals resulting from DNA fragmentations were detected in the cellular nuclei of the bladder within 24 h after SCI (Figure 3A). A distinct line of apoptotic cells was present in the urothelium—specifically, in the umbrella cells layer exposed to urine in the bladder (Figure 3B).

Statistical analysis revealed that the percentage of apoptotic cells significantly increased in the bladder 24 h after injury compared to that of normal animals (Figure 3C). There was a significant increase in the frequency of apoptotic cells in the urothelium in both male and female rats after SCI compared to sham-operated animals (control). However, male rats showed more robust apoptosis than female rats after SCI (Figure 3D).

### 3.4. Severe Pathological Changes Occur during the Chronic Phase after SCI

**Figure 4:**
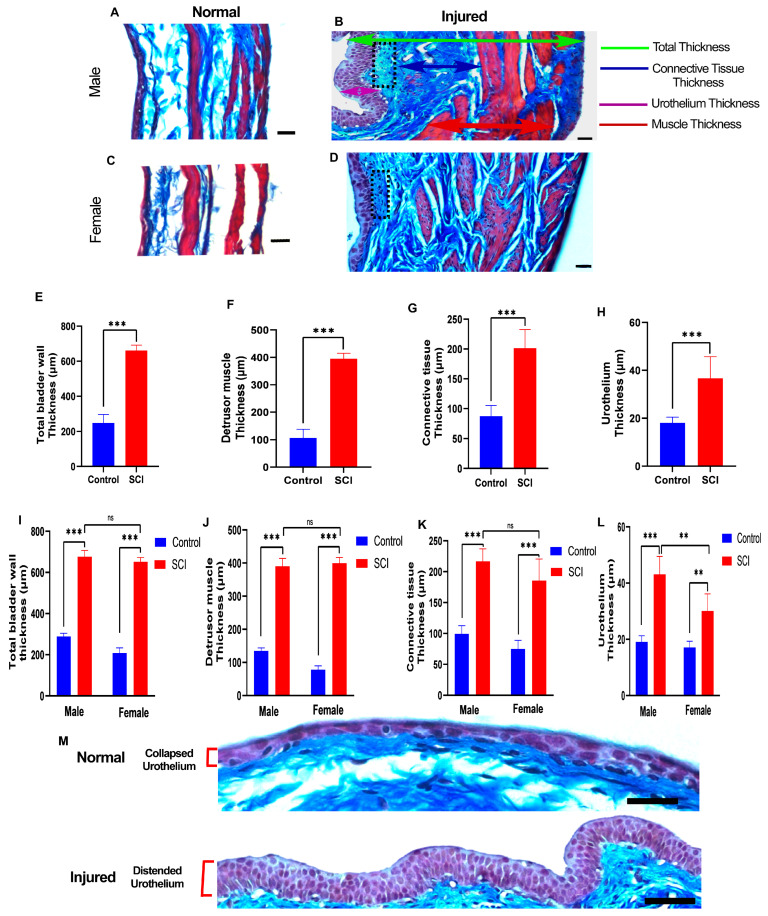
Substantial histological changes in bladder layers are present at 6 weeks after spinal cord injury (SCI). (**A**–**D**) Representative images show transverse sections of the bladder wall of uninjured and injured male and female rats stained by Masson’s trichrome staining. The green arrow shows the total bladder wall thickness, red arrows indicate the thickness of the detrusor smooth muscle layer, blue arrows indicate collagen fibers of connective tissue, and the purple arrow demonstrates urothelium layer thickness. In addition, fibroblasts in the lamina propria layer display high proliferation after SCI (black dotted area) (**B**,**D**). Scale bar = 30 µm; (**E**–**H**) Bar diagrams show a significant effect of injury on the average thickness of the total bladder wall (df = 18, t = 23.56, *** *p* < 0.001, Student’s t test) (**E**), detrusor smooth muscle (df = 18, t = 24.40, *** *p* < 0.001, Student’s t test) (**F**), connective tissue (df = 18, t = 9.969, *** *p* < 0.001, Student’s t test) (**G**), and urothelium (df = 18, t = 6.248, *** *p* < 0.001, Student’s t test) (**H**). (**I**–**L**) Two-way ANOVA (sex x injury) revealed a consistent and significant effect of injury on both males and females regarding the thickness of the total bladder wall (F (1, 16) = 24.95, *p* = 0.0001), detrusor muscle (F (1, 16) = 10.74, *p* = 0.0047), connective tissue (F (1, 16) = 7.703, *p* = 0.0135), and urothelium tissue (F (1, 16) = 13.01, *p* = 0.0024). (** *p* ≤ 0.01, *** *p* ≤ 0.001, ns: non-significant) (**M**) Urothelium in a distended state 6 weeks after injury compared to the collapsed state of control rats. Scale bar = 50 µm. Statistical comparisons were made using a two-way ANOVA, and Tukey’s post hoc test was used for individual group differences. Error bars are the mean ± SD.

Sections of the bladder from the 6-week post-SCI group were stained with Masson’s trichrome stain. Unlike bladder sections from animals shortly after SCI, histological examinations showed substantial changes in all three layers of the bladder, including the urothelium, lamina propria, and detrusor muscle layers (Figure 4A–D), instead of just the umbrella cell layer.

We then compared the thickness of the total bladder wall, detrusor muscle, connective tissue of the lamina propria, and urothelium between the control and SCI-injured rats. Each bladder layer showed significant trends of thickening and, consequently, an increasing total bladder wall thickness compared to control rats (Figure 4E–H).

We compared the control and SCI-injured rats stratified by sex, confirming the same pattern of alterations happening in all three layers in male and female rats in the chronic phase (Figure 4I–L). An increased number of fibroblastic cells were present in the lamina propria layer located beneath the urothelium in the chronic phase (Figure 4B) of both genders. The thickness of the detrusor muscle and connective tissues of the lamina propria did not differ between male and female rats after SCI (Figure 4I–K), but the urothelia of the male rats were significantly thicker than those in female rats after SCI (Figure 4L). However, the transitional urothelia of both male and female rats after injury were distended instead of collapsed in control rats (Figure 4B,D,M).

### 3.5. Robust Neutrophil Infiltration during Hemorrhagic Cystitis in Male Rats

**Figure 5:**
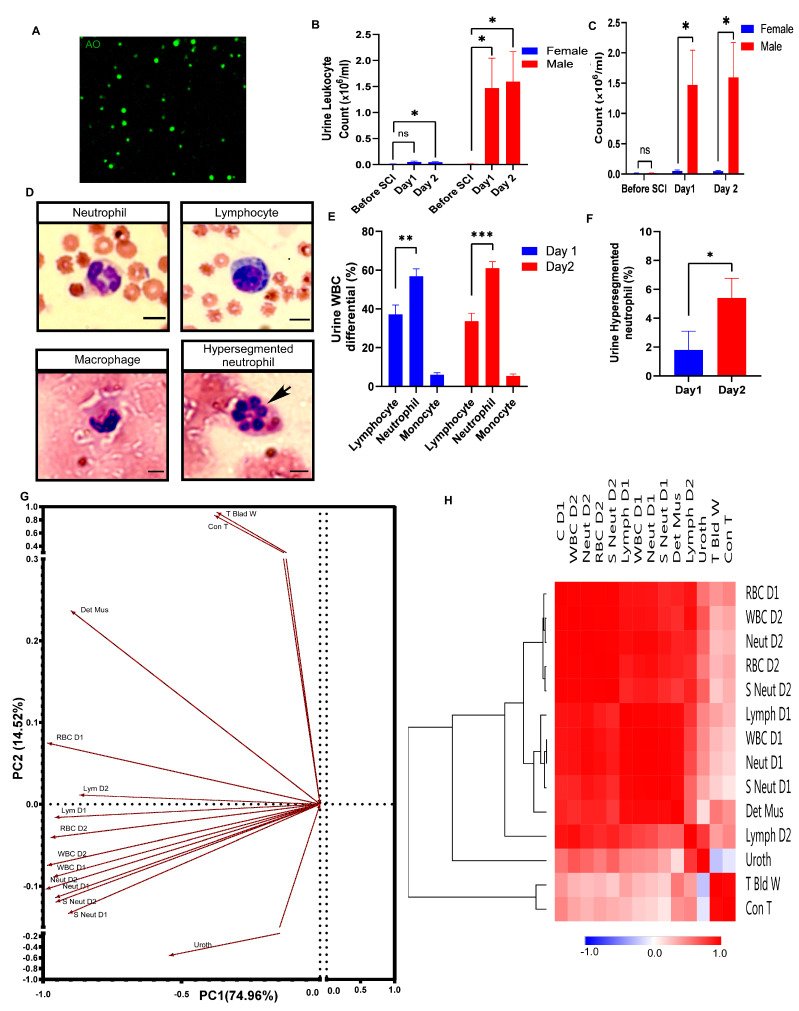
Hypersegmented neutrophil infiltration with a higher number of leukocytes in urine (predominantly neutrophil) in male rats compared to female rats, correlating with the severity of hemorrhagic cystitis. (**A**) Representative images of collected urine samples at 24 h after SCI, stained with the fluorescent dye acridine orange (green) to label leukocytes infiltrating the bladder wall into urine; (**B**,**C**) Bar diagrams indicate the average urine leukocytes count in male and female rats 2 days after SCI compared with that before SCI. Repeated measures ANOVA test (for females comparison: F (1.091, 4.366) = 6.186, *p* = 0.0613; for males comparison: F (1.399, 5.597) = 12.02, *p* = 0.0119) followed by Tukey’s post hoc test (* *p* ≤ 0.05; ns: non-significant) for planned multiple comparison (**B**) or Student’s t tests (before injury: t = 0.6109, df = 8, *p* = 0.5582; day 1: t = 2.474, df = 8, * *p* < 0.05; day 2: t = 2.524, df = 8, * *p* < 0.05) for planned multiple comparison (**C**); (**D**) Wright staining performed on urine samples following SCI differentiated white blood cells (WBC); Hypersegmented neutrophils with five or more nuclear lobes (arrow) Scale bar = 10 μm; (**E**) Bar diagrams show the percentage of the white blood cell differential (lymphocyte, neutrophil, and macrophage) count from male urine samples 1 day and 2 days following SCI by wright staining quantification. ANOVA test (for Day 1 comparison: F (2, 12) = 50.39, *p* < 0.0001; for Day 2 comparison: F (2, 12) = 78.02, *p* < 0.0001) followed by Tukey’s post hoc test for planned multiple comparison. ** *p* ≤ 0.01, *** *p* ≤ 0.001; (**F**) Quantification of the percentage of hypersegmented neutrophils from male urine samples 1 day and 2 days after SCI by Wright staining. More hypersegmented neutrophils are present at 2 days after SCI (df = 4, t = 4.129, * *p* < 0.05, Student’s t test). Error bars indicate SEM; (**G**) Principal Component Analysis (PCA) loading plot based on the urine parameters and bladder histological profiles of the male rats after SCI. The length of the arrows indicates the magnitude of the loading for the variable. Principal components 1 and 2 can represent 90.8% of the variation between samples. Variables include the thickness of the total bladder wall (T blad w), detrusor muscle (Det Mus), connective tissue (Con T), and urothelium (Uroth); urine analysis parameters of day 1 (D1) and day 2 (D2) after injury: total leukocyte count (WBC D1, WBC D2), total neutrophils (Neut D1,Neut D2), total lymphocytes (Lym D1, Lym D2), and total hypersegmented neutrophils (S Neut D1, S Neut D2); severity of hemorrhagic cystitis based on urine red blood cell count (RBC D1, RBC D2); (**H**) A clustered heatmap of Pearson correlation coefficients based on the correlation matrix of pairwise using the Pearson distance and average linkage. Dark red denotes a high correlation (r → 1), dark blue denotes a high anti-correlation (r → −1), and white denotes a lack of correlation (r ≅ 0).

To determine whether the infiltrated leukocytes into the bladder are responding to hematuria after SCI, we stained the leukocytes in urine with fluorescent dyes (acridine orange and propidium iodide) and counted the total number of WBC in the urine of male and female rats (Figure 5A). Male urine leukocytes suddenly increased on day 1 and day 2 after SCI compared to those of the control uninjured rats (Figure 5B). The WBC also rose in the female urine specimens but was statistically significant only on day 2 after SCI (Figure 5B).

Given that sex differences in hemorrhagic cystitis severity were observed after SCI, we asked whether the total leukocyte counts showed a similar pattern of difference in urine white cell counts after SCI. Our results showed robust and significant differences in the leukocyte numbers in urine between male and female rats on both day 1 and day 2 after SCI, even though there was no significant difference between male and female rats before injury (Figure 5C).

We also asked whether the unusually robust number of leukocytes observed in the urine of male rats during severe hematuria might be reflected in WBC differential counts in urine (Figure 5D). On day 1 and day 2 after SCI (Figure 5E), differential WBC counts in urine showed that the percentage of lymphocytes is higher than the percentage of neutrophils in rat blood [32]. When hematuria was present, the percentage of neutrophils was significantly greater than that of lymphocytes in urine on both day 1 and day 2 after SCI (Figure 5D,E).

To assess the neutrophil heterogeneity in urine, we counted neutrophils with different nuclear morphologies (Figure 5D). The Wright-stained urine smear revealed a few neutrophils with a higher number of nuclear lobes (>four lobes) among conventional neutrophils with two to four segmented nuclei in urine in male rats after SCI. The percentage of hypersegmented neutrophils increased significantly in the urine samples on day 2 versus day 1 after spinal cord injury (Figure 5F).

### 3.6. Underlying Trends among the Variables in an Unbiased Multivariate Analysis

Given the profound gross hematuria that occurred in male rats after SCI, we wanted to understand the nature of the association among urine analysis in the acute phase and histology variables in chronic phase in male rats. To do so, we conducted principal component factor analysis (PCA), which allowed us to spot two underlying trends among variables (Figure 5G). This analysis yielded two orthogonal PCs, explaining 89.47% of the total variance among urine analysis and histology variables. Variables related to the urine analysis in the acute phase loaded highly on principal component 1 (PC1), and variables related to the histology, including the total bladder wall and connective tissue thickness, loaded highly on principal component 2 (PC2).

PCA demonstrated that the first principal component (PC1) correlates most strongly with the neutrophil count on day 1, WBC count on day 2, RBC count on day 1, RBC count on day 2, and WBC count on day 1 (loading score: 0.99, 0.985, 0.984, 0.971, and 0.962, respectively); however, the lowest correlation to PC1 was observed with the total bladder wall and connective tissue thickness (loading score: 0.37 and 0.38, respectively). In contrast, the second principal component (PC2) most strongly correlated with histological changes occurring in the chronic phase, including the total bladder wall and connective tissue thickness loading score (0.91 and 0.87, respectively).

To analyze the correlations among variables as well as to visualize the associations among them in the different stages after SCI, hierarchical clustering analysis based on a heatmap matrix of pairwise Pearson correlations of urine analysis and histology variables in male rats was performed (Figure 5H). The heatmap of the clustered correlation matrix revealed a strong correlation of RBC counts on day 1 and day 2 with leukocyte analysis results. The RBC counts on day 1 correlate most strongly with the WBC count on day 2, the neutrophil count on day 2, the hypersegmented neutrophil count on day 2, and the WBC count on day 1 (correlation coefficient: 0.99, 0.985, 0.98, 0.97, and 0.90, respectively). Similarly, the largest correlation coefficient to RBC counts on day 2 was observed with the hypersegmented neutrophil count on day 2, the neutrophil count on day 2, the WBC count on day 2, the neutrophil count on day 1, and the WBC count on day 1 (correlation coefficient: 0.989, 0.988, 0.97, 0.92, and 0.91, respectively). However, the histology variables in the chronic phase in male rats, including the total bladder wall, connective tissue, and urothelium thickness, have the lowest correlation to RBC counts on day 1 and day 2.

As a result, the dendrogram clusters based on the Pearson distance and average linkage using hierarchical clustering analysis (Figure 5H) showed that the RBC counts on day 1 and day 2 have the closest clusters with the WBC count on day 2, the neutrophil count on day 2, and the hypersegmented neutrophil count on day 2. The RBC counts on day 1 and day 2, in contrast, have the most distance from the total bladder wall, connective tissue, and urothelium cluster.

## 4. Discussion

Patients with spinal cord injury may show sustained ulcerations as well as other abnormal alterations of the bladder uroepithelium [6,33], which may result in bladder susceptibility to chronic cystitis or inflammation, possibly related to chronic bacterial infections [7]. Although many articles have reported significant physiological and functional alterations following SCI in the urinary bladder [34,35], the detailed pattern of hemorrhagic cystitis and urothelium ulcerations in the acute phase after SCI is unknown.

Surprisingly, our retrospective data analyses revealed profound differences in the severity of hemorrhagic cystitis between male and female rats in two different strains of rats. Therefore, we used novel urine analysis methodologies in combination with histological study to ascertain the role of pathological changes and immune cells in hemorrhagic cystitis following SCI. Further, we demonstrated a distinctive line of apoptosis in urothelial umbrella cells and robust neutrophil infiltration with hypersegmented neutrophils in urine. These remarkable features point to potentially important factors causing a rapid breakdown and sloughing of the bladder uroepithelium during hemorrhagic cystitis after SCI.

Our findings confirm a previous report [7] regarding hemoglobin in urine (hemoglobinuria) after SCI. We have shown direct evidence for the presence of a significant number of red blood cells and white blood cells, using the unstained method and Wright staining of urine during hematuria after SCI. Additionally, we showed microscopic evidence of isomorphic round erythrocytes combined with crenated forms in urine that indicate the non-glomerular origin of RBC. Finally, our observation of cherry-colored bladders shown by anatomical dissection confirms the non-glomerular origin of RBC or hemoglobin in urine [36].

Importantly, histological examinations of the bladder revealed the robust recruitment of leukocytes to the bladder blood vessels, with alternations in vascular integrity resulting in red blood cell and neutrophil extravasation from inflamed blood vessels early after SCI, followed by the accumulation and dissemination of leukocytes and RBC into the lamina propria and uroepithelium layers. Coincidentally, we observed the disruption of the bladder urothelium layer with the sloughing of urothelium cells, allowing for the penetration of leukocytes and erythrocytes into the bladder lumen and urine.

In agreement with our data, previous studies reported that hemorrhagic cystitis after SCI correlates with rapid disruptions in the uroepithelium and increased water and urea permeability [5]. Additionally, early vascular permeability also occurs in the bladder, as indicated by increased protein and hemoglobin levels in urine just 6 h after spinal cord injury [7]. Interestingly, injured vessels in intact regions of the spinal cord after SCI can originate from the secondary pathogenesis of the inflammatory response [37,38]. Overall, our results indicate that, in addition to affecting the mucosal integrity and its transepithelial resistance, SCI also leads to a rapid disruption of the bladder vascular structure. This manifested in the inflammatory cell accumulation and the breakdown of the endothelial permeability barrier with the extravasation of red blood cells. Therefore, both vascular dysfunction and uroepithelium layer ulcerations are essential pathological features of hemorrhagic cystitis after SCI.

Our retrospective analysis of a macroscopic urine examination of 142 rats from two different rat strains unveiled different patterns of hematuria between male and female rats. Male rats in both strains exhibited significantly more severe hematuria than female rats after SCI. Microscopic examinations of the urine revealed a remarkable increase in erythrocytes in the urine of male rats compared to female rats.

A previous study has also demonstrated significantly longer durations of hematuria in male rats compared to female rats, perhaps because male rats have longer urethras, and clotting may block the urine flow after hematuria [15]. However, we showed by retrospective and experimental studies that longer durations of hematuria are more likely to be a consequence of considerably more severe hemorrhagic cystitis in male compared to female rats. Furthermore, we also found devastating effects of SCI on the bladder urothelium integrity and vascular structure, which exacerbated hemorrhagic ulcers in male rats, potentially requiring more time to recover.

Interestingly, we found a pattern of a fast spontaneous resolution of hematuria in both male and female rats with no therapy. Additionally, the number of urine RBC often fell off quickly in just a few days after SCI, suggesting that the pathological consequences of acute hemorrhagic cystitis differ from more chronic apoptotic changes in the bladder. Our findings are in line with previous studies showing that the early recovery of hematuria signs can occur after bilateral contusion injury [7,15]; however, increased urine protein and the aberrant expression of bladders’ tight junction protein with regions of an incompletely differentiated urothelium can be observed in the chronic phase after SCI [7,8]. Thus, unlike the spontaneous and rapid recovery of hematuria, in which early protective changes in the bladder limit blood leakage into the urine, the long-term effect of hemorrhagic cystitis needs to be considered seriously in chronic SCI.

We identified a clearly delineated layer of apoptotic cells in the uroepithelium layer of the bladder in the acute phase after SCI. Specifically, umbrella cells are undergoing programmed cell death, explaining the rapid breakdown and sloughing of the bladder uroepithelium after SCI. Within the uroepithelium, specialized umbrella cells send processes that cover the luminal surface of the bladder, forming the main barrier that prevents urine’s re-entry into the underlying mucosa and bloodstream [39,40]. While previous articles mentioned a loss of a network of tight junction proteins among umbrella cells after SCI as the main reason for the permeability of the bladder wall in the acute phase [5], our result indicated that the permeability is not just due to the loss of tight junctions but the apoptosis of the umbrella cells. Our results are consistent with previous findings that show the apoptosis and early shedding of superficial umbrella cells in rats and mice with SCI [5,8].

Importantly, we showed a higher percentage of apoptosis in males compared with females in the urothelium layer, which may be related to the higher intensity of hematuria in male rats. Previous studies have shown sex differences in the susceptibility to apoptosis; vascular cells and renal tubular epithelial cells from females are more resistant to oxidative stress-induced apoptosis than those from males [41,42]. These results suggest that the sex disparity in the susceptibility to apoptosis at the uroepithelium layer and the disruption in vascular integrity induced by SCI can result in severe hemorrhagic cystitis in male compared to female rats.

Our bladder morphometric analysis showed that bladder hypertrophy is a result of the thickening of all layers of the urinary bladder. The microscopic evaluation of the bladder wall layers in rats after SCI revealed that increased collagen accumulation, the hypertrophy of detrusor muscles, and the fibrosis of the urothelium contribute to the hypertrophy. Earlier studies [6,43] have shown lamina propria fibrosis and hypertrophy of the detrusor muscle layer in the bladder after SCI.

Rats with chronic SCI may exhibit uroepithelium barrier dysfunction with increases in the urine protein [7]. Both morphological and physiological dysfunctions of bladder permeability are similar between male and female rats in the chronic phase. In humans, uncoordinated contractions of the bladder muscle (detrusor) and bladder-urethral sphincters, sometimes called detrusor–sphincter dyssynergia [44], also lead to a significant neurogenic bladder condition in both male and female patients [45,46]. Bladder wall hypertrophy may contribute to increased outlet resistance.

Our study is also consistent with a previous study [8] reporting that umbrella cells are replaced by small, superficial cells found in the basal and intermediate cell layers with an aberrant expression of keratins and other differentiation markers, indicating incomplete regeneration. Overall, this pattern of dysfunction might contribute to the increased vulnerability to cystitis and bladder cancer that is all too common in chronic SCI patients [47,48,49].

Our finding of greater neutrophilic infiltration and hyper-segmented neutrophils in the bladder associated with more severe hemorrhagic cystitis in male rats is of interest for the following reasons. Male rats have much greater peripheral blood neutrophilia than female rats after SCI (Ayala, et al. in preparation). This may explain the greater severity of hemorrhagic cystitis in male rats. Second, SCI releases a free radical called acrolein [50,51] that is also released by cyclophosphamide [52,53,54] and known to cause hemorrhagic cystitis [55,56,57,58,59], including apoptosis [60].

We demonstrated a profound number of leukocytes in the urine of male rats after SCI in comparison with female rats, with only a mild increase in leukocytes. Interestingly, the WBC differential of male urine samples revealed robust numbers of neutrophils in urine. The presence of neutrophils in urine can explain how the guided recruitment of neutrophils during hemorrhagic cystitis occurs by sequential steps including the sequestration of neutrophils in the bladder vasculature, bladder vascular dysfunction, transendothelial migration into the lamina propria layer, and, subsequently, the transurothelial migration of neutrophils into the bladder lumen and urine. In agreement with our data, which indicate the critical role of neutrophils during hematuria, it has been demonstrated that large numbers of myeloperoxidase (MPO)-immunoreactive neutrophils can be detected in the lamina propria and uroepithelium layers after SCI. Moreover, we showed an increased number of neutrophils on day 2 compared to day 1 post-injury, which is in line with elevated MPO activity by 48 h post-SCI [7]. In addition to the degradation of endothelial junction proteins [61,62], activated neutrophils can induce endothelial cell apoptosis during acute inflammation, resulting in vascular damage [63,64]. While decreased neutrophils infiltration in the bladder after a urinary tract infection has been shown [65], a substantially increased trafficking of neutrophils mobilized from bone marrow was shown in male rats in an ischemia/reperfusion (I/R) injury model [25]. Therefore, activated neutrophils regarding sex discrepancies might be a key determinant for the disruption/destabilization of the bladder vascular endothelium and urothelium early after SCI.

Next, we considered the presence of hypersegmented neutrophils in male urine specimens after SCI. Although there were few hypersegmented neutrophils in the urine samples 24 h after SCI, the percentage of hypersegmented neutrophils in urine increased significantly 48 h after injury. Hypersegmented neutrophils are a separate neutrophil subset that is recruited into the peripheral blood during acute inflammation [66]; however, the origin of neutrophils with a hypersegmented nucleus is still unclear [67]. Moreover, hypersegmented neutrophils could mediate CNS damage in optic nerve crush injury [23], whereas some studies have shown that neutrophils with a more (hyper)segmented nucleus can inhibit T cell responses [68,69]. Hypersegmented neutrophils can also represent other physiological conditions such as vitamin B12 deficiency [70] and neutrophil right shifts during aging that the number of lobes increase [71], but it has been shown that these cells are not older than the neutrophils with a normal nuclear morphology recruited during inflammation, and it is unknown whether hypersegmented neutrophils found in those conditions are the same phenotype as the one found during acute inflammation [66].

In the presented investigations, we used principal component analysis (PCA) to determine the dimensionality and relationships among variables related to the urine analysis findings in the acute phase and histology changes in the chronic phase in male rats. While patterns of multivariate covariation were similar and strong across hemorrhagic cystitis and leukocyte recruitment components, a low magnitude of correlations was observed between chronic histology changes and hemorrhagic cystitis variables. Furthermore, since the leukocyte, neutrophil, and erythrocyte counts were the main variables loading in correspondence with the first principal component, which was mainly loaded by parameters related to the severity of hemorrhagic cystitis, our analysis would suggest that chronic histology changes were not essentially involved in the evaluation processes necessary for determining the severity of hemorrhagic cystitis after SCI. The same pattern of histopathologic findings has been observed in spinal cord-injured patients; no significant correlation was found between histopathological inflammation types and histology changes of bladder layers [6]. It has been shown that, despite the significant difference between male and female rats regarding the hematuria duration after SCI, the onset of bladder voiding reflexes had the same pattern in both genders [15], which may indicate that bladder voiding dysfunction can occur in both sexes with the same severity after SCI, and it could not be the cause of the difference between male and female rats regarding the severity of hemorrhagic cystitis. Moreover, we showed the same pattern of bladder histology. In humans, similar to the animal model, SCI can lead to bladder dysfunction, with a deleterious effect on the storage and voiding functions of the urinary bladder [72]. It needs to be considered that, despite the emergence of a spinal micturition reflex pathway, voiding is commonly inefficient following SCI owing to the disturbance in coordination between bladder detrusor muscles contraction and external urethral sphincter muscles relaxation (detrusor sphincter dyssynergia, DSD) [35]. Thus, chronic phase histology changes in the bladder wall in both genders may be explained by a DSD condition typically seen after SCI.

Hierarchical clustering of the urine analysis and histological change parameters in male rats confirmed the cluster similarities that were seen in the principal component analysis, which revealed that hemorrhagic cystitis severity has the closest cluster with leukocytes and, in particular, neutrophils changes. Additionally, our correlation analysis revealed that hypersegmented neutrophils increases at 48 h after SCI had a significant association with the severity of hemorrhagic cystitis. It has been shown that sympathetic activity after SCI could contribute to acute neutrophil infiltration from the spleen to spinal cord [73]. Although splenic neutrophils could be a source of neutrophils with a hypersegmented morphology [74], it was demonstrated that hypersegmented neutrophils could also be a distinct subset of neutrophils that can be mobilized from bone marrow during inflammation [66,75]. Notably, during acute inflammation, hypersegmented neutrophils have a lower antibacterial capacity than normally segmented neutrophils [76,77], which needs to be considered regarding infection susceptibility in SCI patients and SCI models [78]. Therefore, even though hypersegmented neutrophils might not be the cause of hemorrhagic cystitis, the presence of this subset of neutrophils might be part of the pathophysiological effect of SCI on the immune response.

The most common cause of cystitis is an infection, specifically from bacteria [47], but our microscopic urinalysis and Wright staining did not detect bacteria or parasites in urine. Furthermore, all the rats after SCI were treated for 7 days with broad-spectrum postoperative antibiotics to treat any ongoing infection of the urinary tract. Although viral infection could also develop viral hemorrhagic cystitis [79,80], it can just occur after long-term immunosuppression conditions; however, hemorrhagic cystitis after SCI could be identified 24 h after injury. Thus, immediate hemorrhagic cystitis might be a sterile or non-infectious class of cystitis.

This study involved the characterization of histological bladder changes in the acute phase performed 24 h after SCI; the bladder was not expressed manually for urination. For chronic phase evaluation after SCI, we expressed the bladders manually one time daily in both male and female rats to eliminate urine inside the bladder. Therefore, we prevent a manually expressed bladder effect on the acute phase evaluation, and for chronic phase evaluation, we utilized a similar procedure for males and females. Moreover, spinal cord nerves have control over urethral sphincter function, and a spinal cord injury results in sphincter dysfunction, with increased bladder outlet resistance in both males and females with the same pattern of urine retention with increased intra-bladder pressure. Therefore, further studies on the increased intra-bladder pressure effect on the initiation or augmentation of hemorrhagic cystitis after SCI can advance our knowledge.

Interestingly, it has been shown that an SCI severity-correlated infiltration of lymphocytes SCI may cause hematuria; the incidence of gross hematuria in rats after FTY720 treatment significantly decreased compared to that of the control groups after SCI [81]. We also showed the infiltration of lymphocytes into the bladder and urine in the acute phase after SCI; however, FTY720 treatment can maintain the integrity and functionality of endothelial cells, with a reduction in vascular permeability and significantly decreased neutrophil infiltration [82,83], which are prominent deleterious features we characterized in hemorrhagic cystitis in this study.

## 5. Conclusions

Here, we report that immune cell trafficking can play an important role during hemorrhagic cystitis in male rats following SCI; however, differences in leukocyte recruitment networks, including chemokines and adhesion molecules, in males and females can be considered in future studies [25]. Furthermore, greater neutrophil activation and function have been shown in male rats compared to female rats in a trauma-hemorrhagic shock model [84]. Therefore, it is worth emphasizing that, according to the results of our study, the neutrophil function comparison between males and females, especially for hypersegmented neutrophils, must be considered in SCI models.

In conclusion, we show that more severe hemorrhagic cystitis can occur in male rats compared to female rats after SCI. Furthermore, a distinct line of apoptosis in umbrella cells with disrupted bladder vessels is a profound pathological feature of hemorrhagic cystitis in the acute phase following SCI. Although the exact cause or causes of SCI-induced hematuria are not known, our findings provide insights into the role of immune cells infiltration, especially neutrophils subsets such as hypersegmented neutrophils, during hemorrhagic cystitis in male rats. In addition, bladder histology changes in the chronic phase are not sex-restricted and have a low magnitude of correlation with the severity of hemorrhagic cystitis in male rats. As such, this study opens new doors towards understanding the role of sex after SCI, which might be targeted for bladder dysfunction therapies.

## Data Availability

The datasets used and/or analyzed during the current study are available from the corresponding author upon reasonable request.
